# Cycle Length Restitution in Sinoatrial Node Cells: A Theory for Understanding Spontaneous Action Potential Dynamics

**DOI:** 10.1371/journal.pone.0089049

**Published:** 2014-02-12

**Authors:** Patric Glynn, Birce Onal, Thomas J. Hund

**Affiliations:** 1 The Dorothy M. Davis Heart and Lung Research Institute, The Ohio State University Wexner Medical Center, Columbus, Ohio, United States of America; 2 Department of Biomedical Engineering, College of Engineering, The Ohio State University, Columbus, Ohio, United States of America; 3 Department of Internal Medicine, The Ohio State University Wexner Medical Center, Columbus, Ohio, United States of America; Loyola University Chicago, United States of America

## Abstract

Normal heart rhythm (sinus rhythm) is governed by the sinoatrial node, a specialized and highly heterogeneous collection of spontaneously active myocytes in the right atrium. Sinoatrial node dysfunction, characterized by slow and/or asynchronous pacemaker activity and even failure, is associated with cardiovascular disease (e.g. heart failure, atrial fibrillation). While tremendous progress has been made in understanding the molecular and ionic basis of automaticity in sinoatrial node cells, the dynamics governing sinoatrial nodel cell synchrony and overall pacemaker function remain unclear. Here, a well-validated computational model of the mouse sinoatrial node cell is used to test the hypothesis that sinoatrial node cell dynamics reflect an inherent restitution property (cycle length restitution) that may give rise to a wide range of behavior from regular periodicity to highly complex, irregular activation. Computer simulations are performed to determine the cycle length restitution curve in the computational model using a newly defined voltage pulse protocol. The ability of the restitution curve to predict sinoatrial node cell dynamics (e.g., the emergence of irregular spontaneous activity) and susceptibility to termination is evaluated. Finally, ionic and tissue level factors (e.g. ion channel conductances, ion concentrations, cell-to-cell coupling) that influence restitution and sinoatrial node cell dynamics are explored. Together, these findings suggest that cycle length restitution may be a useful tool for analyzing cell dynamics and dysfunction in the sinoatrial node.

## Introduction

Cardiac pacemaking depends on the tight temporal and spatial synchronization of a specialized population of spontaneously active cells in the sinoatrial node (SAN). Importantly, SAN dysfunction, characterized by asynchronous, irregular pacemaker activity and/or failure is linked to cardiovascular disease and associated with increased mortality in heart failure [1]–[3]. Furthermore, SAN disease is common in the aging population [4]. While great strides have been made in understanding the role of specific ion channels, Ca^2+^ handling proteins, and regulatory molecules in controlling spontaneous SAN cell activity [4]–[7], important questions remain about the nature of SAN dysfunction at the cell and tissue level. At the same time, unlike other cardiac cell types (e.g. atrial and ventricular myocytes), there is a lack of understanding regarding control of SAN cell dynamics.

A restitution relationship has previously been defined to describe myocyte action potential (AP) dynamics in response to pacing [8]–[11]. The restitution hypothesis states that AP duration (APD) depends on the duration of the preceding diastolic interval (DI), with a shorter DI resulting in shorter APD due to decreased recovery time for ion channel gates. The APD restitution curve is determined by pacing the cell to steady state, applying a premature stimulus with progressively shorter coupling interval, and plotting the AP duration as function of preceding DI. A steep restitution curve (slope greater than or equal to one) is associated with increased susceptibility to beat-to-beat alternations in APD (APD alternans). APD alternans, in turn, are thought to be arrhythmogenic by introducing spatial gradients in APD that promote unidirectional conduction block and initiation of reentry [11].

In this study, we hypothesized that irregular SAN cell spontaneous activity reflects an inherent restitution property similar to that responsible for AP dynamics in paced atrial and ventricular myocytes. Here, we define a method for assessing CL restitution in SAN cells and demonstrate the utility of CL restitution in predicting SAN cell dynamics (e.g., the emergence of irregular spontaneous activity). We perform detailed analysis of ionic and structural factors that influence CL restitution in mathematical models of the SAN cell and tissue. Finally, we identify a link between CL restitution curve morphology and SAN behavior at the cell and tissue level. We anticipate that CL restitution will be a useful tool that may be implemented in both experimental and mathematical studies to predict susceptibility to dynamical instability and SAN dysfunction.

## Methods

Ion channel kinetics underlying the SAN AP were simulated using a detailed model of the murine SAN cell [12]. This particular model was selected based on its accurate representation of important features of the murine SAN AP (e.g. morphology, firing rate), facilitating comparison to our previous experimental and modeling studies on SAN dynamics in the mouse [13]–[16]. We expect that, similar to APD restitution, CL restitution will be broadly applicable for understanding SAN dynamics across species. The model was allowed to reach steady-state (20 sec of spontaneous activity) before implementing a perturbation protocol to assess CL restitution. Briefly, a subthreshold conservative stimulus [17] (10 ms duration) is applied at the maximum diastolic potential (MDP) between the last steady-state AP (AP_ss_) and the first perturbed AP (AP_1_). The stimulus amplitude is varied to advance or delay onset of AP_1_ until either phase resetting occurs (for a depolarizing stimulus) or perturbation fails to cause any further change in onset of AP_1_ (for a repolarizing stimulus). The first perturbed CL (CL_1_) is defined as the time interval between AP_1_ and AP_ss_ (time of peak used as reference), while CL_2_ is the time between the second AP following perturbation (AP_2_) and AP_1_. Finally, the CL restitution curve is created by plotting CL_2_ vs. CL_1_ over the entire range of applied stimuli.

Spontaneous activity and termination were also studied in a one-dimensional fiber comprised of 50 mouse SAN cells coupled to 50 mouse atrial cells. A gradient in electrophysiological properties and electrical coupling was introduced in the SAN section, as described [16], to allow for proper activation of the fiber.

Computer code for mathematical models was written in C++ and compiled using Intel Composer XE 2011 for Linux. Computer simulations were performed on a Dell PowerEdge R515 server (Dual 6 core, 32 GB RAM running CentOS-6.2), as described [14]–[16], [18], [19].

## Results

**Figure 1:**
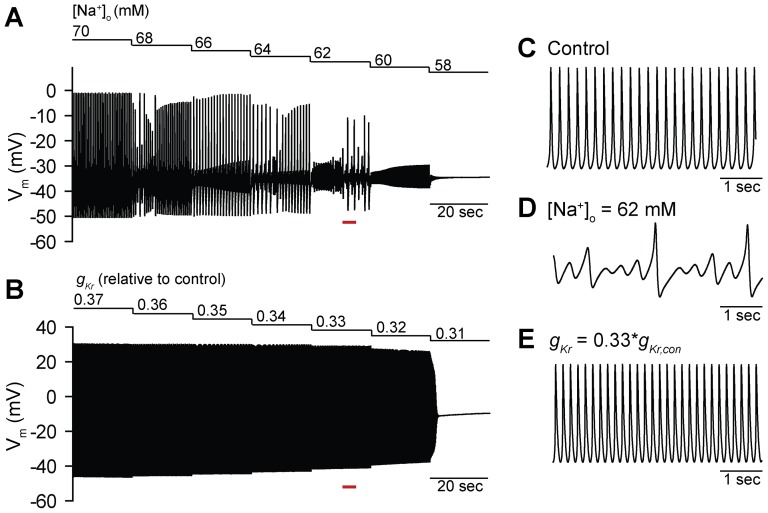
Termination modes of spontaneous activity in a mathematical model of the mouse sinoatrial node cell. (**A**) Irregular activation and periodic skipped beat runs are apparent prior to termination as [Na^+^]_o_ is decreased stepwise from 70 mM to 58 mM. (**B**) In contrast, a gradual decline in regular activity occurs as *g_Kr_* is decreased from 0.37 to 0.31 times its control value. (**C-E**) Representative spontaneous action potentials under (**C**) control conditions, and during stepwise decrease in (**D**) [Na^+^]_o_ and (**E**) *g_Kr_* (corresponding time periods marked by *red bars* in panels **A** and **B**).

Previous experimental and computational studies have identified distinct modes of termination in SAN cells in response to perturbation (e.g. decreased [Na^+^]_o_, L-type Ca^2+^ channel block) [20]–[24]. Similarly, our previous computational and experimental studies have demonstrated the emergence of complex irregular activity preceding termination of spontaneous activity in ankyrin-B-deficient mouse SAN cells [13], [16]. Furthermore, we demonstrated that irregular activity at the single cell level translated to a shift of the primary pacemaker site and even failure in a detailed two-dimensional model of the intact sinoatrial node [16]. Based on these findings, we sought to determine the mechanism(s) underlying emergence of irregular activity and termination of spontaneous activity in SAN cells. We first examined the response of SAN spontaneous activity to two perturbations previously shown to elicit very different dynamic behaviors: 1) Decreased extracellular [Na^+^] ([Na^+^]_o_) [20], [24], and 2) block of the rapid delayed rectifier K^+^ current *I_Kr_* (Figure 1) [20], [23]. Consistent with previous reports [20], [24], we observed the emergence of irregular activity and skipped beats preceding termination of spontaneous activity as [Na^+^]_o_ was decreased in a stepwise fashion from 70 to 58 mM. In contrast, spontaneous action potentials gradually declined in amplitude until activity terminated as the maximal conductance of *I_Kr_* (*g_Kr_*) was decreased from 0.37 to 0.31 times its control value (Figure 1). While previous studies have applied tools from nonlinear dynamics (e.g. bifurcation diagrams, Poincare plots) to describe these behaviors, the underlying mechanism remains unclear [20]–[22], [25]. Furthermore, the field lacks an effective method for efficiently determining the inherent dynamical stability of a given cell.

**Figure 2:**
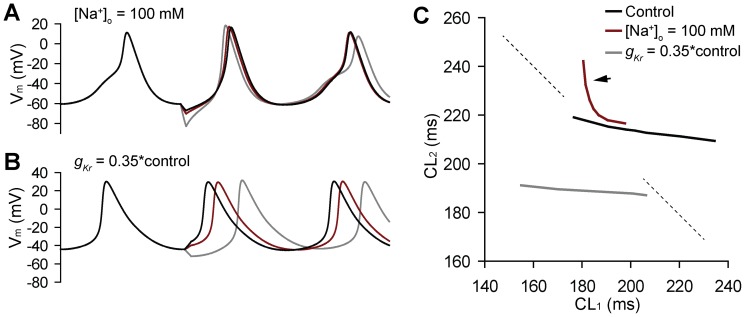
The cycle length restitution curve. (**A–B**) Simulated spontaneous action potentials during a protocol to determine the cycle length restitution curve. A 10-ms stimulus is applied with varying amplitude at the maximum diastolic potential to accelerate or delay the subsequent spontaneous action potential. Time between 2^nd^ and 1^st^ APs following perturbation (CL_2_) is then plotted as a function of time between 1^st^ perturbed and steady-state APs (CL_1_). (**C**) CL restitution curves for control (*black*), low [Na^+^]_o_ (*red*) and *I_Kr_* block (*gray*). Note that low [Na^+^]_o_ results in a curve with an abrupt transition from a relatively flat region to a very steep region (maximal slope >>−1 indicated by *arrow*; *dashed line* has slope of −1 for reference).

We hypothesized that SAN cells possess an inherent CL restitution property that may be assessed using the newly defined perturbation protocol (described in Methods). Furthermore, we predicted that the slope of the CL restitution curve might provide important predictive information regarding the likely mode of termination of spontaneous activity (irregular activity vs. gradual decline) in response to perturbation. To test this hypothesis, we determined CL restitution curves for the baseline model (control) and for two different conditions: low [Na^+^]_o_ and *I_Kr_* block, which promote termination through irregular activity and gradual decline, respectively (Figure 2). We specifically chose a value for [Na^+^]_o_ (100 mM) that was well above the threshold for irregular activity (threshold ∼68 mM, see Figure 1) to test whether the restitution curve could be used as a predictor of susceptibility to irregular activity and mode of termination. Consistent with our hypothesis, the restitution curve was uniformly flat (maximal slope >−1) for control conditions and in the presence of *I_Kr_* block. In contrast, low [Na^+^]_o_ produced a curve with an abrupt transition from a relatively flat region to a very steep region (maximal slope <<−1, indicated by *arrow* on red curve in Figure 2). These simulations indicate that the CL restitution curve provides important information regarding the inherent dynamical stability of the cell and, potentially, the mode of termination.

**Figure 3:**
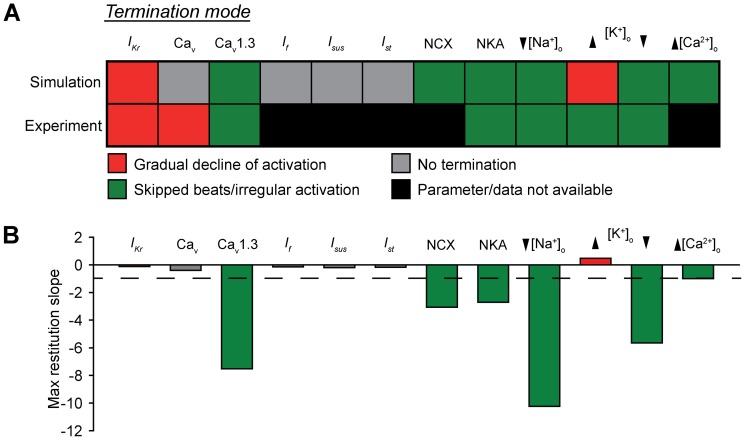
Parametric analysis of termination modes and restitution curve slope. (**A**) Unless otherwise indicated, each model parameter was decreased stepwise from its control value until spontaneous activity terminated or the value reached zero. For ionic currents (e.g. *I_Kr_*), the affected parameter was the maximum channel conductance. For the Na^+^/Ca^2+^ exchanger (NCX) and Na^+^/K^+^ ATPase (NKA), affected parameters were scaling factor (kNaCa) and maximum current, respectively. Altogether data were collected from 500 independent simulations. Experimental data is provided for comparison where available (compiled from [20], [23], [24]). (**B**) Maximal CL restitution curve slope in the model for each parameter just before onset of irregular activity, termination, or value reaches zero. Color of bar corresponds to mode of termination identified in **A**. Note that there is good agreement between parameters that produce skipped beats and result in steep restitution (indicated by *green* in **A** and **B**). Dashed line corresponds to a slope of −1.

To determine the robustness of the CL restitution curve as a predictor of termination mode, we performed a detailed parametric analysis in the model (Figure 3). Select parameters were incrementally changed from their control values until spontaneous activity was terminated or the parameter value was zero. Steady-state (200 seconds) spontaneous activation was determined for each parameter set. Resulting termination modes were compared to experimental reports in the literature, where available. In general, we found good agreement between model and experiment (agreement in 5 of 7 instances where comparison was possible), with irregular activity being the more common mode, accounting for 75% (6 of 8 cases) and 71% (5 of 7 cases) of termination in model and experiment, respectively (Figure 3A). We next used the model to determine the maximal slope of the CL restitution curve for each case at a parameter value just above the threshold for termination (in the case of gradual decline to termination) or onset of irregular activity (Figure 3B). In every instance, parameters that promote irregular activity en route to termination also produce steep CL restitution curve (maximal slope <−1, Figure 3). Conversely, parameters that fail to terminate (even at value of zero) or promote termination via gradual decline result in relatively flat return maps (maximal slope >−1). It is also interesting to note that, in general, loss of repolarizing current favors shallow restitution and gradual decline of activity (e.g. *I_Kr_* block, elevated [K^+^]_o_) while loss of depolarizing current/gain of repolarizing current favors steep restitution and skipped beats/irregular activity (e.g. Ca_v_1.3 block, NCX block, decrease in [Na^+^]_o_). These results support our central hypothesis that the slope of the CL restitution curve may be used as a predictor of likely termination mode. Furthermore, these findings suggest that by manipulating CL restitution it may be possible to alter SAN cell dynamics.

**Figure 4:**
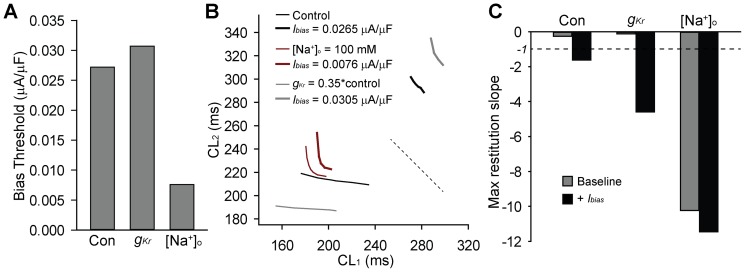
Effect of bias current on cycle length dynamics. (**A**) Bias current threshold to induce irregular activity in control, *I_Kr_* block (*g_Kr_* = 0.35*control), or low [Na^+^]_o_ (100 mM). (**B**) CL restitution curves and (**C**) maximal slope for control, *I_Kr_* block, and low [Na^+^]_o_ at baseline and during bias current injection.

While studies thus far demonstrate the utility of restitution in analyzing SAN dynamics at the level of the single cell, cardiac pacemaking depends on coordinated activity of a well-organized collection of electrically coupled SAN cells. As a first step in extrapolating our analysis to the multi-cellular pacemaker complex, we sought to determine the effect of a constant (bias) repolarizing current (as an approximation of electrical loading) on restitution and spontaneous activity in the control model and with *I_Kr_* block or low [Na^+^]_o_ (100 mM, above the threshold for spontaneous irregular activity) (Figure 4). Interestingly, in all three cases, we identified a threshold bias current that led to irregular activity/skipped beats with the lowest threshold corresponding to low [Na^+^]_o_ and highest threshold for *I_Kr_* block (Figure 4). We next generated CL restitution curves at baseline and in the presence of the bias current just below the threshold for induction of irregular activity (Figure 4). In all cases, a repolarizing bias current led to a steeper curve compared to baseline (Figure 4B–C). We also tested whether a depolarizing bias current would have the opposite effect on the slope of the restitution curve. Indeed a depolarizing bias current (amplitude  = −0.01 mA/mF) flattened the curve reducing the max slope from −0.27 to −0.15 in the control model and from −10.92 to −1 under low [Na^+^]_o_ conditions (not shown; we were unable to assess for *I_Kr_* block as spontaneous activity terminated even for depolarizing bias current <<−0.01).

**Figure 5:**
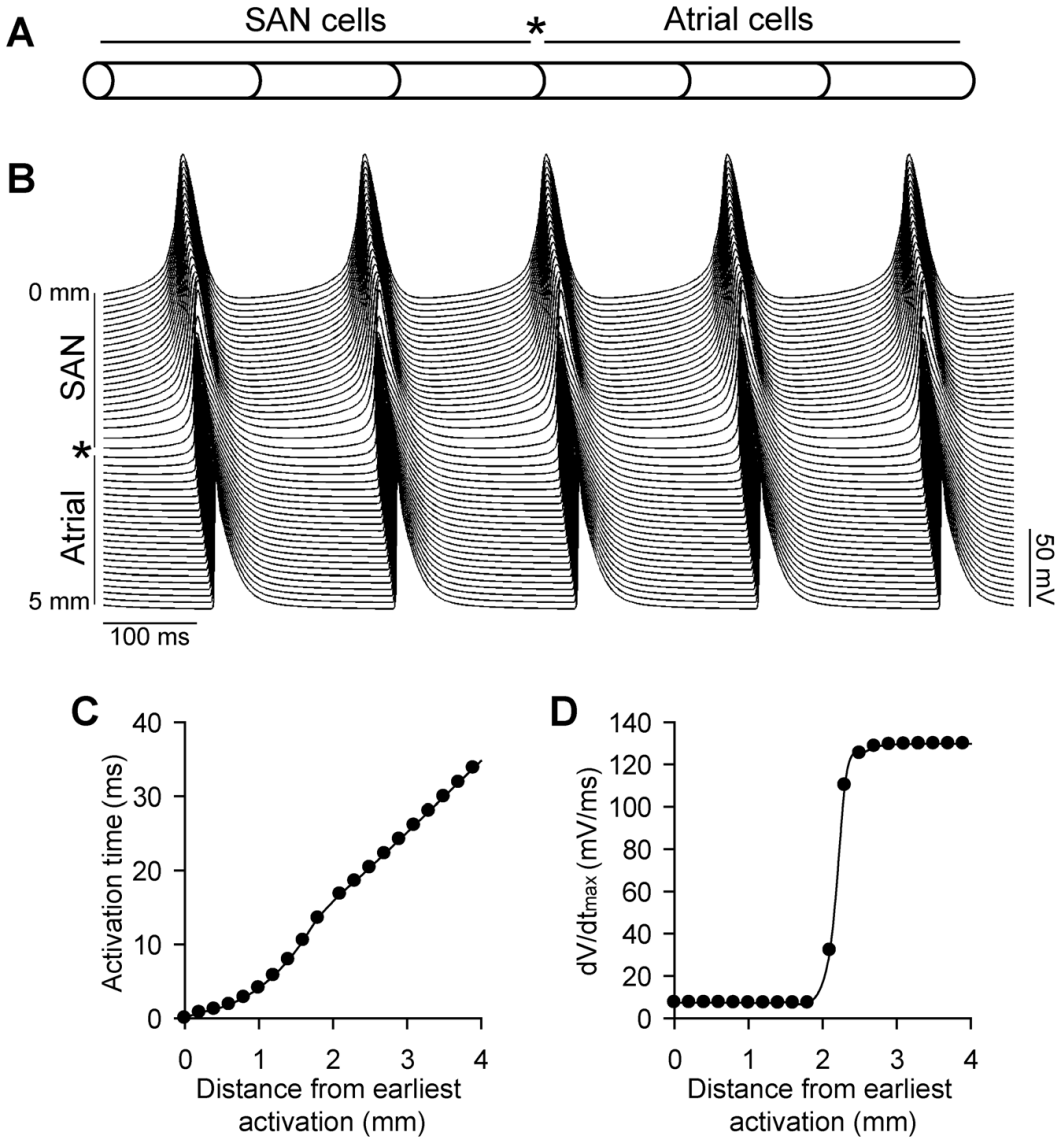
One-dimensional sinoatrial node fiber model. (**A**) 50 mouse SAN cells are electrically coupled to 50 mouse atrial cells to create a one-dimensional fiber. (**B**) Spontaneous action potentials along the fiber during five consecutive cycles at steady-state. (**C**) Activation time and (**D**) dV/dt_max_ along the fiber during one cycle at steady-state.

**Figure 6:**
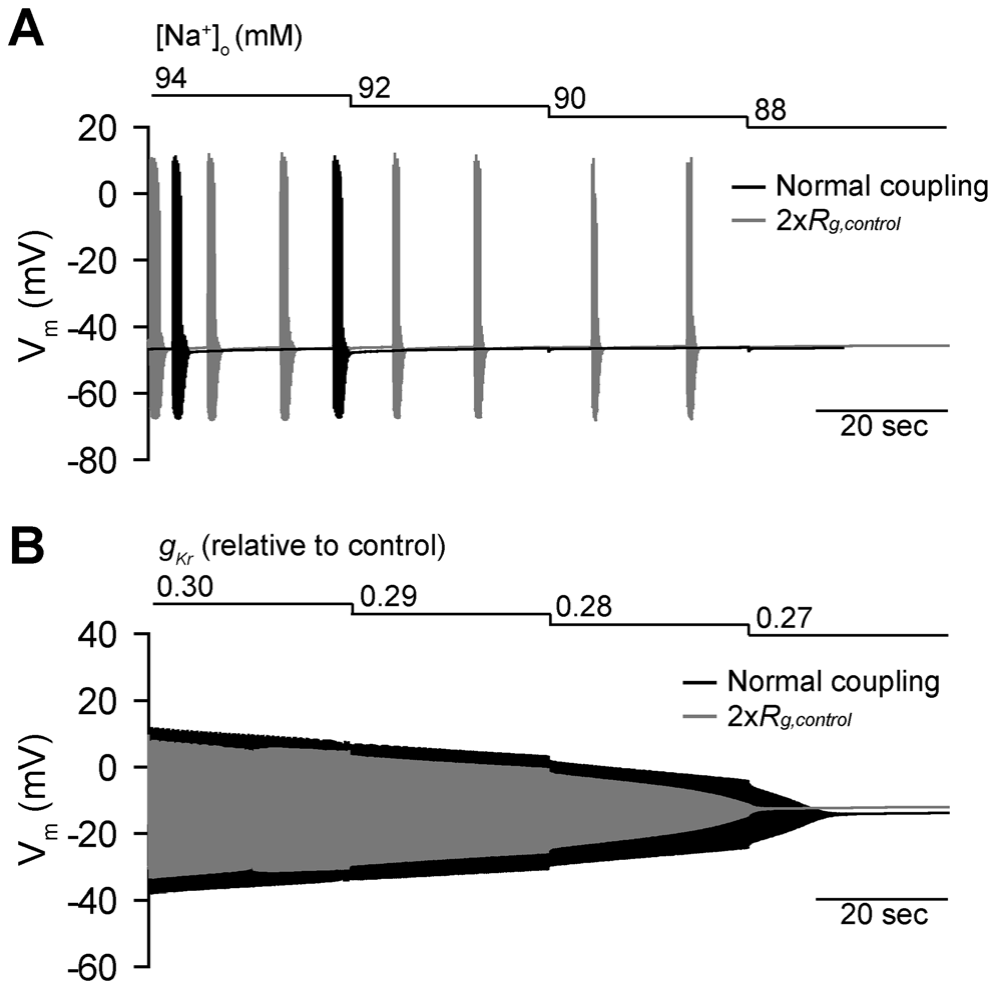
Termination of spontaneous activity in the one-dimensional fiber model. (**A**) [Na^+^]_o_ or (**B**) *g_Kr_* was decreased in a stepwise fashion under normal coupling conditions (*black*) and with uniform gap junction uncoupling (twofold increase in gap junction resistance, *gray*) to reduce electrotonic loading of sinoatrial node cells. Termination of spontaneous activity occurs earlier in the fiber than in the single cell as [Na^+^]_o_ decreases, preceded by irregular activation and long runs (up to 20 sec) of skipped beats. Furthermore, uniform gap junctional uncoupling (*gray*) delays termination. In contrast, termination is delayed in the fiber relative to the single as *g_Kr_* is decreased (gradual decline) and uncoupling accelerates termination.

Based on these findings, we hypothesized that conditions associated with steep restitution at the level of the single cell (e.g. low [Na^+^]_o_) would show increased susceptibility to irregular activity/termination in the context of the coupled tissue where coupled cells experience electrotonic loading (analogous to repolarizing bias current). To test this hypothesis, we implemented a one-dimensional fiber of SAN cells coupled to atrial cells (Figure 5). We first systematically decreased [Na^+^]_o_ or *g_Kr_* in the SAN region (similar to protocol outlined in Figure 1) to determine the termination mode and threshold in the coupled tissue (Figure 6). As expected from simulations with repolarizing bias current, both onset of irregular activity and termination of spontaneous activity occurred earlier (higher [Na^+^]_o_) in tissue compared to the single cell ([Na^+^]_o_ = 92 mM for termination in tissue compared to 58 mM for single cell). In contrast, the termination occurred later (more block) for *g_Kr_* in tissue compared to single cell (*g_Kr_* = 0.27*control for termination in tissue compared to 0.31*control in single cell). We also sought to determine what effect uniform uncoupling would have on termination in the fiber. Increasing gap junction resistance two-fold in the SAN region delayed termination for low [Na^+^]_o_ (termination at 88 mM rather than 92 mM, Figure 6A) but slightly accelerated termination for *I_Kr_* block conditions (*g_Kr_* = 0.28*control rather than 0.27, Figure 6B). Together, these findings suggest that the CL restitution curve is useful not only for predicting single cell dynamics but also behavior at the level of the intact tissue. Namely, cells demonstrating steep restitution and/or irregular activity at the single cell level are likely more susceptible to activation failure in the context of the intact tissue.

**Figure 7:**
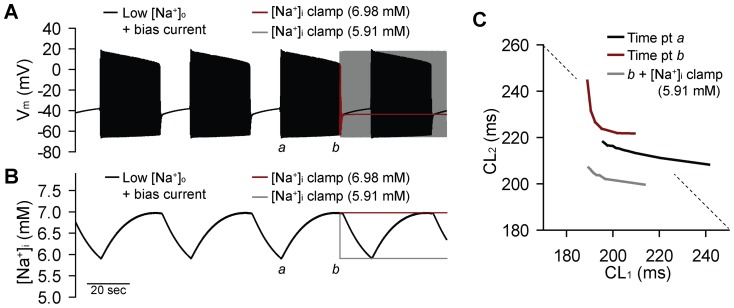
Ionic mechanism for irregular SAN activity with long pauses. (**A**) Simulated spontaneous APs and (**B**) [Na^+^]_i_ from a single SAN cell subjected to low [Na^+^]_o_ (100 mM) and constant, low amplitude (0.0077 µA/µF) bias current stimulation. During AP firing, [Na^+^]_i_ rises until a threshold is reached (∼6.98 mM) at which point spontaneous activation terminates and [Na^+^]_i_ slowly falls until a second threshold is reached (∼5.91 mM) and the pattern repeats. Clamping [Na^+^]_i_ to the termination threshold (*red line* in **A** and **B**, clamp applied at time point labeled *b*) results in complete termination of activity, while clamping to the recovery threshold (*gray line*) eliminates the skipped beat runs resulting in regular periodic activity. (**C**) The CL restitution curve was determined for the model with low [Na^+^]_o_ and bias current stimulation just after onset of regular periodic activity (*black line*, determined at time point labeled *a* in panels **A** and **B**) or prior to onset of skipped beat run (red line, determined at point marked *b*). [Na^+^]_i_ was then reset to the low threshold value and restitution was determined again at the same time point (*gray line*). The restitution curve from the control model (normal [Na^+^]_o_, no bias current) is shown for reference (*black line*). Dashed line denotes a slope of −1. [Na^+^]_i_ alters the slope of the restitution curve with much steeper slope at higher [Na^+^]_i_.

In addition to the effect of coupling on the termination threshold, we observed an interesting impact on the overall activation pattern. While irregular activity at the level of the single cell may be characterized by higher order periodic patterns and intermittent skipped beats (see Figure 1A), in the tissue we observed intervals of regular activity interspersed with long pauses (Figure 6A). To determine the mechanism for this distinct type of activity, we returned to the single cell with low [Na^+^]_o_ (100 mM) and bias current stimulation (0.0077 µA/µF, just above the previously determined threshold for irregular activity). Consistent with the irregular pattern in the coupled fiber, we observe long skipped beat runs in the single cell model with low [Na^+^]_o_ and bias current injection (Figure 7). We hypothesized that this behavior reflected the existence of system “memory” in addition to the beat-to-beat dynamics captured by the CL restitution (first order approximation for restitution is that activity depends only on preceding CL). We further hypothesized that, similar to short-term memory effects observed in ventricular myocytes [26], [27], SAN memory depends on intracellular ion concentration changes that typically occur with a slower time course than other AP processes (e.g. ion channel gating). Interestingly, we find that during the period of regular activity, intracellular [Na^+^] ([Na^+^]_i_) slowly increases until activity terminates allowing [Na^+^]_i_ to gradually decrease until activity is restored and the pattern repeats itself (Figure 7B). [K^+^]_i_ shows a similar, but inverted, pattern (increases when Na^+^ decreases and vice versa, not shown). The central role of [Na^+^]_i_ in governing this behavior is demonstrated by clamping [Na^+^]_i_ to either the recovery threshold (e.g. value at time point *a* in Figure 7A–B) or the termination threshold (value at time point *b*) during spontaneous activation (in both cases, clamp applied at time point *b*). Clamping [Na^+^]_i_ to the termination threshold (higher value) resulted in complete termination of spontaneous activity (*red lines* in Figure 7A–B). Conversely, clamping [Na^+^]_i_ to the recovery threshold resulted in uninterrupted periodic activity (*gray lines* in Figure 7A–B). In contrast, implementation of a similar clamp with [K^+^]_i_ had very little effect on activation pattern (not shown).

We hypothesized that [Na^+^]_i_ regulates SAN cell dynamics by altering CL restitution. To test this hypothesis, we determined restitution at two different points during the irregular periodic activity shown in Figure 7: 1) just after onset of regular periodic activity (time point *a* in Figure 7A–B corresponding to low [Na^+^]_i_) and 2) just before onset of a long skipped beat run (time point *b*). We observed a dramatic change in the maximal slope of the restitution curve between the two time points. Specifically, at the onset of periodic activity, the curve is relatively flat (max slope >−1), while just before termination, the curve demonstrates an abrupt transition from a flat to a very steep region (max slope <<−1). We also measured restitution just before termination of activity but with [Na^+^]_i_ reset to its value just after onset of activity. We found that lowering [Na^+^]_i_ in this manner both shifted and flattened (max slope >−1) the restitution curve. Together these simulations demonstrate that [Na^+^]_i_ affects CL restitution and SAN cell dynamics with [Na^+^]_i_ accumulation favoring a steep restitution and irregular SAN activity. Under conditions where the restitution curve shows an abrupt transition from a flat to very steep region, (see Figures 2C and 7C), [Na^+^]_i_ promotes a distinct type of SAN activity characterized by regular activity periodically interrupted by long skipped beat runs.

## Discussion

**Figure 8:**
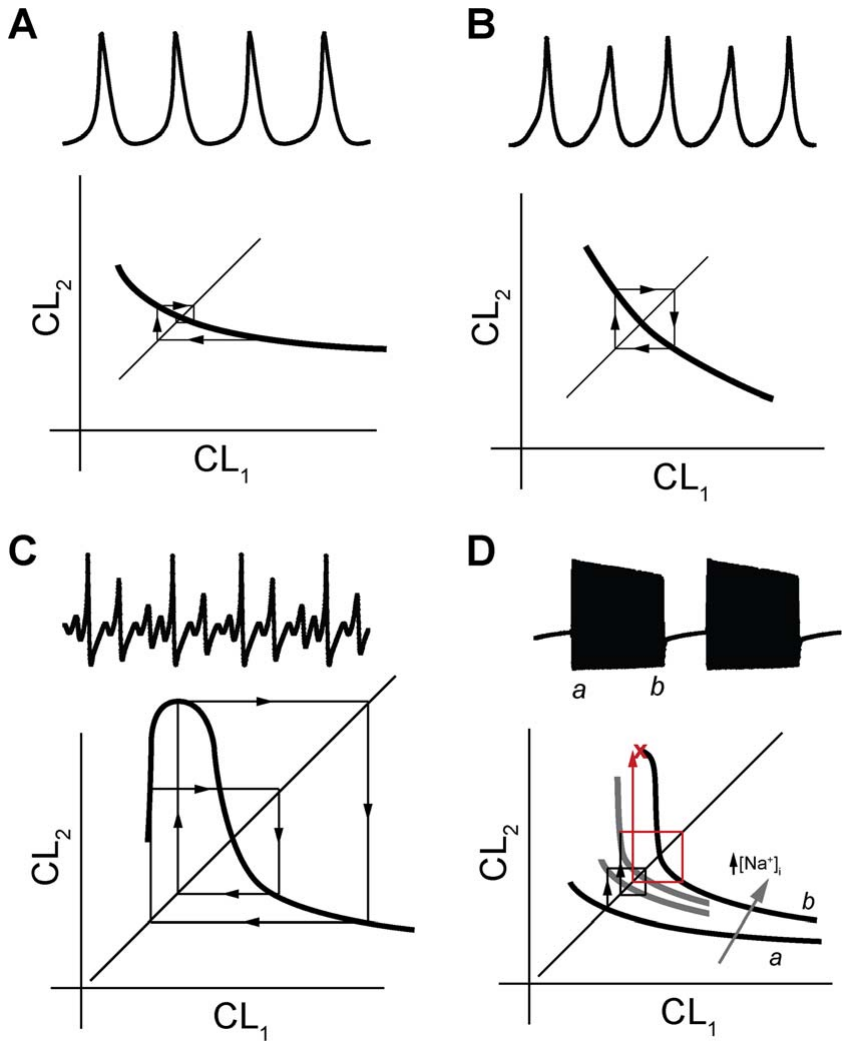
A graphical method for understanding the relationship between spontaneous SAN activity and restitution. Simulated spontaneous APs are shown in *top* of each panel with schematic representation of corresponding restitution curve in *bottom*. The identity line may be superimposed on the restitution curve to create a return map for tracking CL dynamics in response to a perturbation (*arrows* demonstrate iterative response to perturbation from fixed point, defined as intersection of return map with identity line). (**A**) Regular periodic activity (control model) occurs when restitution slope is shallow (slope >−1). Perturbation from steady-state results in eventual return to stable fixed point. (**B**) 2:2 periodic behavior (APs correspond to control model with bias current  = 0.0274 mA/mF) results from a monophasic curve with a slope equal to the critical value of −1 (in this example, CL stably alternates between 306 ms and 283 ms). (**C**) Higher dimensional periodic activity (e.g. 4:4) and skipped beats may result from a multiphasic curve with regions of steep slope (<−1) (APs correspond to [Na^+^]_o_ = 63 mM), (**D**) irregular activity with long skipped beat runs may occur in instances where the CL restitution curve experiences an abrupt transition from a shallow region (slope<−1) to a very steep region (slope>>−1) (APs are shown for [Na^+^]_o_ = 100 mM, bias current  = 0.0077 µA/µF). Schematic curves and corresponding return map trajectory are shown as spontaneous activity progresses from time point *a* (onset of activity) to *b* (just before termination). In this case, dynamic changes in [Na^+^]_i_ may produce intermittent long skipped beat runs by shifting the curve and altering its slope.

While it is well-established that SAN pacemaking depends on rhythmic activity of membrane ion channels and calcium cycling proteins at the level of the single cell, the governing dynamics responsible for regulating spatial and temporal control of SAN synchrony remain elusive. Research in this area has become increasingly reductionist and while this approach has yielded gains in understanding the underlying mechanism for monogenic disease, tremendous controversy remains regarding the factors that govern the regular heart rhythm [28]. Nonlinear dynamics approaches are well-suited to the study of a complex system such as the cardiac pacemaker. In fact, previous studies in this area have generated important insights into SAN cell dynamics and have laid the foundation for the SAN restitution theory outlined here [20]–[22], [25], [29]. This previous research has described the different types of SAN spontaneous activity, associated phase space diagrams, and role of major ion channels/cell factors (e.g. vagal stimulation) in regulating dynamics. Here, we advance this work by using an analytical and computational approach to generate a number of testable predictions regarding the dynamic behavior of SAN cells. Our major findings include: 1) the CL restitution curve provides valuable information about the dynamical status of the SAN cell, is relatively straightforward to assess, and may be used to make predictions about likely termination mode and behavior in coupled tissue; 2) CL restitution gives rise to a wide range of irregular dynamical behavior from beat-to-beat alteration (CL alternans) to regular periodic activity interspersed with extended periods of quiescence (long skipped-beat runs) (different classes of activity are illustrated in Figure 8); 3) Electrotonic loading of cells in the intact tissue (similar to repolarizing bias current stimulation in a single cell) alters SAN cell dynamics, in part, by increasing the slope of the CL restitution curve; 4) Single cell conditions favoring relatively steep CL restitution are associated with increased susceptibility to irregular activity and even termination in the coupled tissue; and 5) Similar to other cardiac cell types (e.g. ventricular myocytes [27]), SAN cell dynamics demonstrate “memory” as a result of intracellular ion changes (particularly Na^+^) that alter restitution.

SAN CL restitution serves as a clear analogue to APD restitution in quiescent myocytes (e.g. atrial and ventricular myocytes). Similar to APD restitution, CL restitution reflects a fundamental dependence of cell activity on preceding recovery time. APD and CL restitution both assume that activity depends only on events in the preceding cycle, a first order approximation that does not account for system memory due, for example, to relatively slow intracellular ion changes. Finally, in both cases, restitution is straight forward to assess and interpret. We anticipate that CL restitution will serve a valuable role in advancing our understanding of SAN dynamics and dysfunction, similar to the proven utility of APD restitution in analyzing AP dynamics and arrhythmias [27].

There are a number of implications from our findings. First, our analysis predicts that electrical coupling has a dramatic impact on cellular dynamics and that this effect (either palliative or detrimental) depends, in part, on baseline restitution properties of the individual cell. This is a critical factor to consider in light of recent discussion regarding the safety and efficacy of anti-arrhythmic agents that target gap junctions to increase electrical coupling [30], [31]. Our findings suggest that care should be taken in how these drugs (e.g. rotigaptide) are administered as in certain cases such a drug would be predicted to exacerbate sinus node dysfunction. It is important to note the difference between uniform electrical uncoupling studied here and heterogeneous uncoupling/cell loss (e.g. due to apoptosis and/or fibrosis), which in previous studies we have shown to be without exception detrimental to SAN function [14]–[16]. Clearly, loss of spontaneously active cells within the SAN will disrupt the source-sink balance between the SAN and surrounding atrial myocardium, independent of restitution. Another important implication of our findings is that elevated [Na^+^]_i_ promotes steep restitution and irregular dynamics, providing a potential mechanistic explanation for SAN dysfunction closely associated with certain types of cardiac disease (e.g. atrial fibrillation, heart failure). Finally, in a general sense, our studies highlight the importance of considering SAN dynamics (rather than just steady-state CL, for example) when evaluating single cell behavior.

It is important to note important limitations of our study, which uses a computational approach to develop and test a specific hypothesis regarding SAN restitution and dynamics. Going forward, it will be essential to experimentally validate model predictions. Specifically, important future experiments in SAN cells will determine: 1) whether the protocol outlined here may be efficiently implemented to assess restitution *in vitro*, *in situ*, and/or *in vivo*; 2) whether the restitution curve is indeed useful in predicting dynamics and termination mode at cell and tissue level; and 3) whether restitution may be used to better understand cardiac pacemaking mechanisms and dysfunction. It is also important to note that while our study examines the effects of changes in electrical coupling on SAN dynamics in coupled tissue, these changes are likely less important than the inherent differences in coupling in the intact SAN where impedance changes dramatically from the central SAN to the right atrium. Going forward it will be important to consider the functional impact of electrical and structural heterogeneity within the intact SAN on restitution and overall dynamics.
